# The use of misoprostol before hysteroscopy in Nulliparous women: a systematic review and meta-analysis of randomized controlled trials

**DOI:** 10.1186/s12884-024-06993-z

**Published:** 2024-11-27

**Authors:** Noha Salah, Ahmed Mohamed Maged, Safaa I. Mahmoud, Nehal Bassiouny, Reham A. Mohsen, Suzi AbdelAziz, Wael S. Ragab

**Affiliations:** 1 Department of Obstetrics and Gynecology, Kasr Al-Ainy Hospital, Cairo University, Cairo, Egypt; 2 Department of Obstetrics and Gynecology, Fayoum University, Fayoum, Egypt; 3 11 Eid Mostafa Street, Haram, Giza 12111 Egypt

**Keywords:** Misoprostol, Hysteroscopy, Nullipara

## Abstract

**Objectives:**

To assess the value of misoprostol intake before hysteroscopy in nulliparous women.

**Search strategy:**

Databases screening was done from inception to July 2023 using “Misoprostol” AND “Hysteroscopy” AND “Nullipara” and their MeSH terms as keywords.

**Selection criteria:**

Thirteen studies were included in our analysis. Seven studies compared misoprostol to placebo, 3 studies compared it to dinoglandin, 1 study compared it to diclofenac and 4 studies compared different misoprostol doses and routes. These studies were conducted on 1528 participants,958 of them received misoprostol, 221 received dinoglandin, 51 received diclofenac and 308 received placebo.

**Data collection and analysis:**

Extracted data included study place, participants number, inclusion and exclusion criteria, intervention details as dose, route, timing and comparotor, and hysteroscopy details.

**Main Results:**

Ease of cervical dilatation was reported in 3 studies (309 participants) and revealed an effect estimate mean difference (MD) of -0.57 [-1.72, 0.58] and a P value of 0.33. The time needed for cervical dilatation was reported in 6 studies (512 participants) and revealed a MD of -22.96 [-43.29, -2.62] and a P value of 0.03. The preoperative cervical width was reported in 4 studies (263 participants) and revealed MD of 1.69 [-0.09, 3.46] and a P value of 0.06. The number of women with failure of cervical dilatation or who needed further dilatation was reported in 4 studies (372 participants) and revealed a MD of 0.40 with [0.13, 1.17] 95% CI and a P value of 0.09. The preoperative pain was reported in 3 studies (351 participants) and revealed a MD of -0.56 [-2.30, 1.18] and a P value of 0.53. Total number of cases who experienced side effects and procedure complications were reported in 2 and 3 studies (249 and 252 participants) respectively and revealed an effect estimate Odd Ratio of 1.99 and 0.42 with [0.27, 14.67] and [0.14,1.32] 95% CI and a P value of 0.50 and 0.14 respectively. In the 3 studies comparing misoprostol to dinoglandin, The ease of cervical dilatation, time needed for cervical dilatation and preoperative cervical width were evaluated in 1,3 and 2 studies with 60, 436 and 376 participants respectively. The estimated MD were not estimated, 0.17 and 0.01; 95% CI were not estimated, [-4.70, 5.05], and [-0.78, 0.79]; P values of 0.94, 0.98 and 0.99 and I_2_ of 96%,95% and 74% respectively.

**Conclusion:**

Misoprostol improved the time needed for cervical dilatation without affecting the rate of complications or drug side effects when compared to placebo but has similar outcomes to dinoglandin with higher side effects.

**Registration number:**

CRD42023438432.

**Supplementary Information:**

The online version contains supplementary material available at 10.1186/s12884-024-06993-z.

## Introduction

Although TVU can visualize most uterine conditions, its accuracy is questionable [1] and blind D&C may miss a small lesion. The use of hysteroscopy allowed direct visualization of the uterine cavity and the performance of a directed biopsy. It allows both the diagnosis and treatment of most intracavitary lesion and currently, it is considered as the gold standard procedure for evaluation of the uterine cavity in both premenopausal and postmenopausal women [2].

The hysteroscopic outpatient procedure is an established diagnostic test in evaluation of women with abnormal genital bleeding and those with reproductive difficulties without the need for anaesthesia [3].

With the development of instruments and technology, many of the operative procedures can be conducted as outpatient ones with or without the use of local anaesthesia. Although outpatient hysteroscopy is safe and convenient, the uterine instrumentation could be associated with pain [4] and anxiety [5].

Pain is commonly associated with passing the instruments through the cervix as women with narrow cervical os as nullipara and menopausal women have a higher risk of pain and failed procedure than women with wider cervical canal [6].

Several strategies were suggested to decrease pain and anxiety during hysteroscopy. These include pharmacological ones as the use of analgesics, antispasmodics, anti-inflammatory, local anaesthtics, cyclooxygenase-2 inhibitors and opioids and non pharmacological ones as the use of warm distension media, transcutaneous electrical nerve stimulation, music and hypnosis [7].

Most of the complications of hysteroscopy -especially in nullipara – as cervical tears, bleeding and creation of false tracts are linked to cervical dilatation. Cervical preparation before hysteroscopy was suggested to minimize these complications. Different interventions are used for cervical preparation such as osmotic dilators and prostaglandins [8].

Nullipara and postmenopausal women are more susceptible to experience pain and other complications of hysteroscopy as these women have less elastic and less dilated cervical os [9].

Misoprostol is a relatively safe, cheap, readily available synthetic prostaglandin E1 analog that could be taken through various routes including oral, through mucous membranes (vaginal, rectal, and sublingual) and even intrauterine [10].

Misoprostol has many uses in both obstetrics and gynecology. It can be used for prevention and treatment of postpartum hemorrhage after both vaginal and cesarean deliveries (CD) [11], minimizing intraoperative and post operative bleeding during CD [12, 13] and in cervical preparation before uterine instrumentation as IUD insertion especially in high-risk women.

The use of misoprostol may be associated with side effects that are usually mild. These include fever, nausea, vomiting, diarrhea, abdominal pain, dyspepsia and less commonly vertigo, weakness and lethargy. More severe side effects are less common and include hypotension, sinus tachycardia, myocardial infarction, cervical lacerations, pulmonary embolism, anaphylaxis, and thrombosis [14].

Many studies evaluated the use of misoprostol before hysteroscopy in various populations as nullipara, menopausal women [15], women with previous CD with contradictory results. Some studies proved its efficacy in cervical dilatation prior to hysteroscopy and other ones failed to confirm its efficacy [16].

The controversial results of the studies that evaluate the use of misoprostol before hysteroscopy clarify the need for searching the evidence of its use especially in high-risk women as nullipara.

The aim of this systematic review is to assess the efficacy and safety of misoprostol administration before hysteroscopy in nulliparous women.

## Materials and methods

This study followed a prospectively registered protocol (CRD42023438432) at PROSPERO following PRISMA guidelines.

### Eligibility criteria, information sources, search strategy

PubMed central, Scopus, Web of Science, Google scholar, the Cochrane and clinical trial registration databases were searched independently by 2 authors (AM, NS) using the terms “Misoprostol” AND “Hysteroscopy” AND “nullipara” and their MeSH as keywords from inception to July 2023 without language limitation. Data were also searched for in the reference lists of related clinical and review articles, the citation lists of linked publications, abstract of gynecological endoscopy conferences. Incomplete and/or unclear data were clarified through direct contact with the authors.

### Study selection

We included all randomized controlled studies that involved preoperative administration of misoprostol before hysteroscopy in nulliparous women. All studies that compared misoprostol to placebo, dinoglandin drugs or misoprostol at different time or route and all routes of administration including vaginal, oral, or sublingual were included. The inclusion of the studies were selected by 2 authors independently (AM, WS) and any disagreement between the 2 authors were discussed with other authors. Subgroup analysis was carried out for different comparators, doses, routes, and timing of administration. Non-randomized trials, case reports, review articles and editorial opinions were excluded from our review.

### Data extraction

Data extraction was done independently by 2 authors (NB and WSR). The extracted data included centers and country of the trial conduction, masking nature of the study, participants number and characteristics, intervention details (comparator, misoprostol dose, route and time of preoperative administration) and outcomes of the study. The evaluated outcomes included the ease of cervical dilatation and time needed for it, failure of dilatation, preprocedural cervical diameter, preoperative pain score, drug side effects and procedure complications.

### Assessment of risk of bias and quality of evidence

The Cochrane Handbook of Systematic reviews recommendations that included random sequence generation, allocation concealment, blinding of included population, blinding of outcome evaluator, selective data reporting and other biases (as prospective trial registration and funding details) was used to assess the risk of bias of the included studies.

The GRADE system that included the number of trials included in analysis, trials risk of bias, inconsistency, indirectness, imprecision and publication bias was used to assess the quality of evidence.

### Data synthesis

The continuous and dichotomous data were analyzed through calculation of the mean difference and odd ratio with their 95% confidence interval (CI) analysis respectively. The effect size was calculated through the random effect model. I^2^ statistic test was used to estimate heterogeneity of the included studies. A P-value < 0.05 and I^2^ > 40% were set as significant [17]. Analysis was done using the Review Manager (RevMan) version 5.4.1 (The Nordic Cochrane Centre, Cochrane Collaboration, 2020, Copenhagen, Denmark).

## Results

### Study selection

**Fig. 1:**
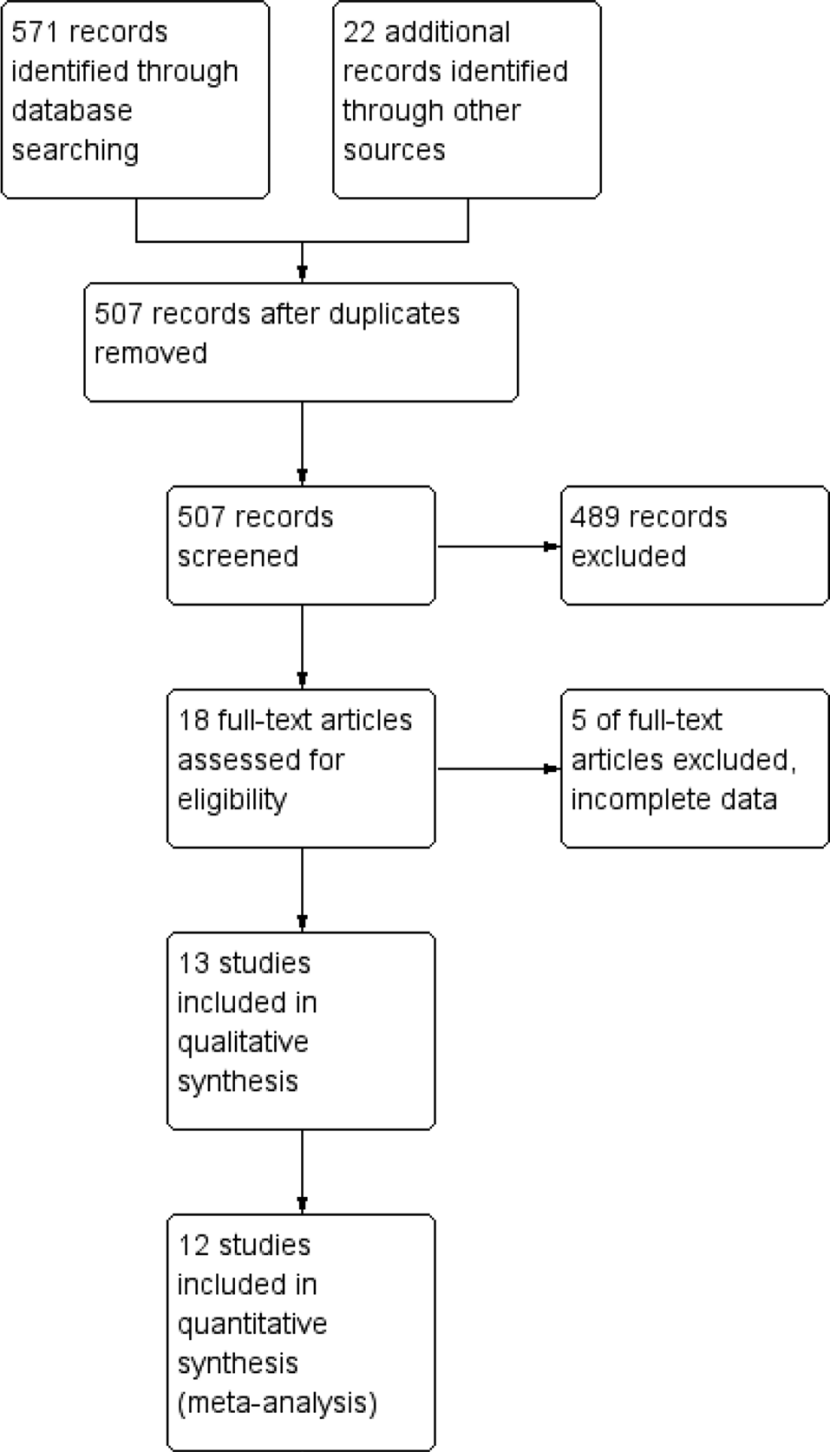
Prisma flow diagram

The Prisma flow chart is shown in Fig. 1.

### Study characteristics

Table 1 describes the extracted data of the included studies and their characteristics.

**Table 1:** Characteristics of the included studies

Study	settings	Design	Size	Participants	Intervention	Outcome	Registration	Funding
Abulnour 2018	Single center Egypt	Open label	66	Inclusion criteria: Nullipara indicated for DH (abnormal HSG, thick endometrium, suspected Müllerian anomalies or infertility. Exclusion criteria: Women with previous delivery (vaginal or CS), previous cervical dilatation and curettage, or lesions (as tears or polyps), Contraindications for prostaglandin (as allergy, bronchial asthma, glaucoma, hypotension, diabetes hepatic and cardiac diseases).	Study group (*n* = 33) 400 ug of vaginal misoprostol inserted 6 h before hysteroscopy. Comparative group (*n* = 33) 3 mg of vaginal dinoprostone inserted 6 h before hysteroscopy. A 5-mm office hysteroscope was used to view the uterine cavity for 20–30 s maximum. No anesthesia.	Easiness of entry score (Likert) Pain at the end of the procedure (VAS). Side effects	No	None
Bakas 2012	Single center Greece	Open label	110	Inclusion criteria: nullipara, premenopausal women fit for DH. Indications for DH included 3 failed IUI; 1 or more failed IVF/ICSI; menometrorrhagia; and intrauterine pathology (suspected by ultrasound or HSG) Exclusion criteria: contraindications to prostaglandins; previous cervical procedures (previous dilatation and curettage, biopsy or excision); and contraindications to hysteroscopy (as bleeding, pregnancy, active infection, or suspicion of malignancy	Group A (*n* = 39) received 200 µg of oral misoprostol at 12 and 6 h before DH; Group B (*n* = 36) received 200 µg of vaginal misoprostol 12 h before DH Group C (*n* = 35) received 200 µg of vaginal misoprostol 4 h before DH.	Preoperative cervical dilation and the need for cervical dilatation. Time needed for dilation, Complications (as uterine or cervical injuries or intrauterine bands), Misoprostol side effects.	No	None
Batukan 2008	Single center Turkey	Double blind	77 (nullipara40)	Inclusion criteria Non pregnant premenopausal women Exclusion criteria: contraindication to PGs (severe asthma, glaucoma, preexisting diseases as hypertension, heart, or kidney), previous cervical procedures or incompetence, significant uterovaginal Prolapse.	Intervention group (*n* = 39, 19 nullipara) 400 mg of oral misoprostol 10–12 h before operative hysteroscopy Comparative group (*n* = 38, 21 nullipara) 400 mg of vaginal misoprostol 10–12 h before operative hysteroscopy A 9 mm rigid resectoscope and 30°forward-oblique lens was used. Distension media 1.5% glycine solution at an insufflation pressure of 100– 150 mm Hg under GA	Preoperative cervical width, Need for cervical dilatation, Time for cervical dilatation, Time for the procedure, complications, and side effects	No	None
Bisharah 2003	Single center Canada	Double blind	40	Inclusion criteria: nulliparous reproductive-age women requiring operative hysteroscopy Exclusion criteria: Not discussed	All participants received 3.75 mg intramuscular leuprolide acetate 4 weeks before hystroscopy Misoprostol group (*n* = 20) 100 ug sublingual misoprostol 12 h before *operative hysteroscopy*. Control group (*n* = 20) sublingual placebo 12 h before *operative hysteroscopy.* a continuous flow resectoscope under GA	Preoperative cervical diameter Time needed to dilate the cervix to 9 mm. The difficulty in cervical dilatation. Side effects complications (cervical lacerations, uterine perforation, false tract, bleeding).	No	None
Fouda 2016	Single center Egypt	Double blind	120	Inclusion criteria: premenopausal nullipara indicated for office hysteroscopy Exclusion criteria: Allery or contraindication to misoprostol (asthma, glaucoma, renal failure, hypertension, and severe heart disease), Pregnancy, severe vaginal bleeding, PID, previous cervical operation, endocervical lesions, and treatment with GnRH agonists.	Long interval group (*n* = 60) 400 ug vaginal misoprostol inserted 12 h and 2 tablets placebo were inserted 3 h before DH. Short interval group (*n* = 60) 2 tablets placebo were inserted 12 h and 400 ug vaginal misoprostol inserted 3 h before DH. A rigid 2.9-mm hysteroscope with a 30° forward oblique lens and an outer sheath diameter of 5 mm Distension media normal saline with pressure between 60 and 100 mm Hg. Hysteroscopy was diagnostic using non-touch (vaginoscopic) technique	pain during and 30 min after the procedure (VAS), , the ease of passing the hysteroscope, complications and misoprostol side effects	NCT02316301	None
Hassa 2013	Single center Turkey	Double blind	152	Inclusion criteria Women with 1ry infertility indicated for DH. Exclusion criteria: Allergy or contraindication to misoprostol (cardiac and/or vascular disease, hypertension, severe asthma, glaucoma, renal failure, contraindication to hysteroscopy ( cervical stenosis, genital infection, vaginal bleeding, genital malignancy, or pregnancy; Allergy or contraindication to NSAIDs (known gastroesophageal disease); history of labor or abortion.	Group 1 (*n* = 51) received 200 mg vaginal misoprostol 6 h and rectal placebo 1 h before DH Group 2 (*n* = 50) received vaginal placebo 6 h and 100 mg rectal diclofenac sodium rectally 1 h before DH. Group 3 (*n* = 51) received vaginal placebo 6 h and rectal placebo 1 h before DH. A rigid 30-degree 4-mm hysteroscope was used without anesthesia or analgesia. The uterine cavity was distended using normal saline at a pressure of 100 to 120 mm Hg	Pain during the procedure (VAS), Time of procedure , patient acceptance (Likert Scale), Need for postprocedural analgesics, and vasovagal symptoms as nausea, vomiting, bradycardia, hypotension, sweating, and syncope.	No	None
Healey 2007	Single center Canada	Double blind	64 (11 Nullipara)	Inclusion criteria: healthy premenopausal women, aged 19 years or more candidate for DH. Exclusion criteria: Allergy to prostaglandins, seizure disorder, or liver disease with abnormal liver functions	Study group (*n* = 33, 7 nullipara) was given 400 ug oral misoprostol 12 h before the procedure. Control group (*n* = 31, 4 nullipara) 50 mg oral B6 (placebo) 12 h before the procedure. Diagnostic hysteroscopy 6 mm was carried out under GA	pre-procedural cervical width, Need for additional dilatation, time required for dilatation side effects complications	No	None
Inal 2015	3 centers Turkey	Double blind	90	Inclusion criteria: infertile Nullipara with no contraindication for hysteroscopy. Exclusion criteria: Allergy or contraindication to prostaglandins (hypertension, severe asthma, heart disease, glaucoma, renal failure, or uncontrolled diabetes); genital infection; previous cervical incompetence or procedures as dilatation curettage, loop electrosurgical excision, or cryotherapy; previous GnRH agonist treatment	Study group (*n* = 30) received 400 ug of vaginal misoprostol 6–8 h before hysteroscopy. Dinoprostone group (*n* = 30) received 10 mg of vaginal dinoprostone 6–8 h before hysteroscopy. Control group (*n* = 30) received vaginal placebo (Lactobacillus acidophilus) 6–8 h before diagnostic hysteroscopy. A rigid standard hysteroscope with an outer sheath measuring 5.5 mm in diameter and a scope with a 30° viewing angle. distension with a saline solution under pressure at 100–125 mm Hg under GA.	Need for cervical dilatation. Preoperative cervical width, duration of dilatation, ease of dilatation, side effects, and complications.	NCT01620814	None
Mohamed 2020	Single center Egypt	Double blind	198	Inclusion criteria Nullipara aged 20–50 years old, indicated for hysteroscopy for infertility, recurrent miscarriage or abnormal uterine bleeding Exclusion criteria Uterine abnormality that would obviate passage of a catheter, cervical stenosis, recent pelvic disease, uterine bleeding. Contraindications to prostaglandins.	Long interval misoprostol group (*n* = 66): received 400 ug vaginal misoprostol 12 h, Two vaginal placebo (folic acid 500 mg) 6 h and 3 h before hysteroscopy. Intermediate interval misoprostol (*n* = 66): received 400 ug vaginal misoprostol 6 h, Two vaginal placebo 12 h and 3 h before hysteroscopy. Short interval misoprostol (*n* = 66): received 400 ug vaginal misoprostol 3 h, Two vaginal placebo 12 h and 6 h before hysteroscopy. A 5.5 mm, 30 degree fibro optic lens rigid hysteroscope with constant uterine distention had by 3 L volume saline bags to dual infusion tubing with a pressure of 150-200mmHg. without the use of anesthesia or analgesia,	Pain immediately after the procedure (VAS). Ease of entry of the cervix (Likert) Baseline width at the beginning of the procedure. The bleeding during the procedures. Time of procedure, and complications	No	None
Nair 2020	Single center India	Double blind	100	Inclusion criteria: premenopausal nulliparous woman aged between 18 and 45 years indicated for office hysteroscopy. Exclusion criteria: active genital infection, ongoing vaginal bleeding, previous cervical surgery, or allergy to misoprostol or clotrimazole	Misoprostol group (*n* = 50) received 200 ug vaginal misoprostol 4 h before the procedure Control group (*n* = 50) received vaginal placebo (clotrimazole) 4 h before the procedure A 3.2‑mm office hysteroscope using distension with normal saline through vaginoscopy technique.	Ease of the procedure (Likert) Time of the procedure Time of cervical dilatation Need for cervical dilatation. Pain during the procedure (VAS) Patients’ satisfaction Side effects complications	CTRI/2019/04/018458	None
Preutthipan 2000	Single center Thailand	Double blind	152	Inclusion criteria: Infertile women with suspected intrauterine abnormalities by ultrasonography, HSG or sonohysterography Exclusion criteria: Patients with early pregnancy, genital tract infection, and normal hysteroscopic findings	Misoprostol group (*n* = 73) received 200 ug vaginal misoprostol 9–10 h before operative hysteroscopy Control group (*n* = 79) received vaginal placebo (a lactose filler) 9–10 h before operative hysteroscopy Diagnostic hysteroscopy was done with a 5.5-mm rigid hysteroscope with a diagnostic sheath. Operative procedures were done using either a 7-mm operative sheath or a resectoscope with an outer sheath 9 mm in diameter.	Preoperative cervical width, need for cervical dilatation, Time of cervical dilatation to 6 and 7–9 mm , Time of the procedure, complications, and side effects	No	None
Preutthipan 2006	Single center Thailand	Double blind	310	Inclusion criteria: Infertile women with suspected intrauterine Abnormalities Exclusion criteria: Patients with early pregnancy, and genital tract infection.	Misoprostol group (*n* = 152) received 200 ug vaginal misoprostol 9–10 h before operative hysteroscopy Dinoprostone group (*n* = 158) received vaginal 3 mg dinoprostone 9–10 h before operative hysteroscopy Diagnostic hysteroscopy was done with a 5.5-mm rigid hysteroscope using carbon dioxide as a distension media. Operative procedures were done using either a 7-mm operative sheath or a resectoscope with an outer sheath 9 mm using 1.5% glycine solution as a distension media.	cervical width at hysteroscopy, Need for cervical dilatation Time of cervical dilatation to 6 and 7–9 mm , Time of the procedure, complications, and side effects	No	None
Tasma 2017	3 centers Netherland	Double blind	149	Inclusion criteria: All postmenopausal and premenopausal nulliparous women with an indication for office hysteroscopy Exclusion criteria: Age < 18 years old, inadequate command of the Dutch language, allergy for misoprostol, previous cervical surgery and active infection	Misoprostol group (*n* = 74) received 400 ug oral misoprostol 12 and 24 h before Hysteroscopy Control group (*n* = 75) received oral placebo 12 and 24 h before hysteroscopy A 5.5-mm rigid hysteroscope using saline infusion at a uniform pressure of 80–100 mmHg as a distension media	Pain before first dose 24 h prior to hysteroscopy; after taking second dose 12 h before the hysteroscopy; immediately after the hysteroscopy (VAS) Patients’ satisfaction Complications Easiness score	No	St Antonius Hospital Investigational Funds

Thirteen studies were included in our analysis [18–30]. These studies were conducted on 1528 participants, 958 of them received misoprostol, 221 received dinoglandin, 51 received diclofenac and 308 received placebo. Three studies were conducted in Egypt [18, 22, 26], 3 in Turkey [20, 23, 25], 2 in Canada [21, 24], 2 in Thailand [28, 29] and 1 study in each of the following countries Greece [19], India [27], and Netherland [30].

All the studies were double blinded except 2 studies [18, 19] and all were conducted in a single center except 2 studies [25, 30] that were conducted in 3 centers. In 7 studies misoprostol was compared to placebo [21, 23–25, 27, 28, 30], in 3 studies misoprostol was compared to dinoglandin [18, 25, 29], in 4 studies it was compared to itself through different routes [20] or different time of [19, 22, 26] while in 1 study misoprostol was compared to diclofenac [23]. The route of administration was vaginal in 10 studies 18–20,22,23,25–29] and sublingual in 1 study [21]. The dose of misoprostol was ranged from 100 ug (1 study) [21], 200 ug (5 studies) [19, 23, 27–29] to 400 ug in 7 studies [18, 20, 21, 24–26, 30]. The timing of administration ranged between 3 h and 24 h before the procedure.

Four studies have 3 arms [19, 23, 25, 26] and the other 9 studies have 2 arms only.

Hysteroscopy was done under general anaesthesia in 6 studies [20, 21, 24, 25, 28, 29] and with no anaesthesia in 6 studies [18, 22, 23, 26, 27, 30] (in 1 study, the use of anaesthesia was not clear and the authors did not clarify it in response to repeated trials of contact [19]). The distension media was saline in 6 studies [22, 23, 25–27, 30], CO2 in 2 study [28, 29], Glycine in 3 studies [20, 28, 29] and not reported in the other 4 studies. In Preutthipan studies in 2000 and 2006, CO2 was used for diagnostic procedure and Glycine 1.5% was used for operative one. Only 3 studies were registered [22, 25, 27].

### Risk of bias of included studies

**Fig. 2:**
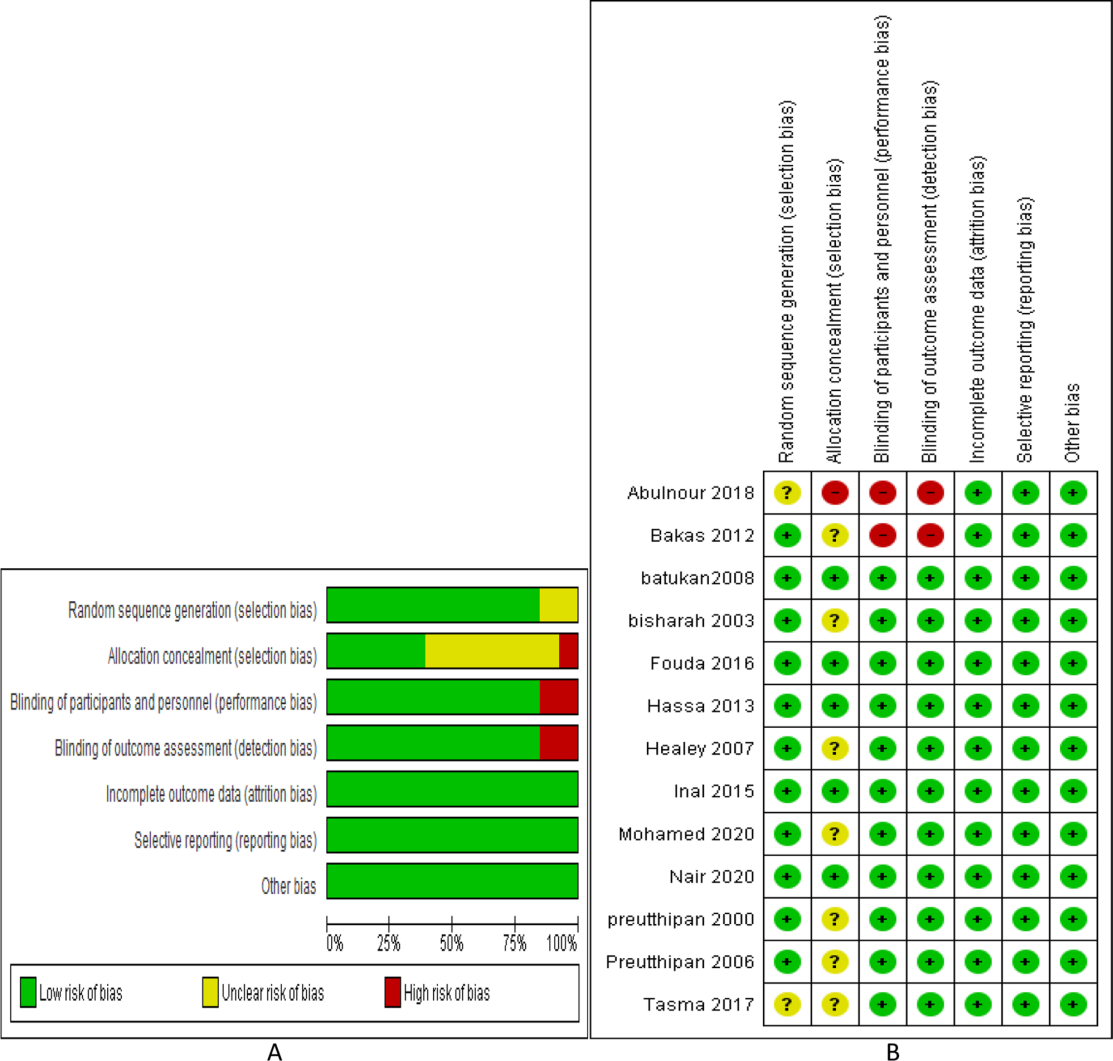
Risk of bias **A** graph and **B** summary

Figure 2; Table 2 describe the risk of bias (graph and summary) and GRADE quality of evidence respectively.

**Table 2:** GRADE quality of evidence

Outcome			No studies	Risk of bias	Inconsistency	Indirectness	Imprecision		Publication bias	Quality
							Sample size	Wide CI		
Ease of dilatation			3	N	S	N	N	S	N	Low
Time for cervical dilatation			6	N	N	N	N	S	N	Moderate
Preoperative cervical width			4	N	N	N	N	S	N	Moderate
Failure to dilate cervix or need further dilatation			4	N	N	N	N	S	N	Moderate
Side effects			2	N	S	N	S	S	N	Low
Specific side effects		Nausea	3	N	N	N	N	S	N	Moderate
		Vomiting	3	N	N	N	N	S	N	Moderate
		Diarrhea	1	N	S	N	S	S	N	Very Low
		Pain	3	N	N	N	N	N	N	High
		Bleeding	3	N	N	N	N	N	N	High
Complications			3	N	N	N	N	S	N	Moderate
Specific complications	Cervical laceration		3	N	S	N	N	S	N	Low
	False tract		2	N	N	N	N	S	N	Moderate
	Uterine perforation		2	N	N	N	N	S	N	Moderate
Preoperative pain			3	N	S	N	N	N	N	Moderate

CI Confidence Interval; N Not serious; S Serious

### Synthesis of results

Misoprostol versus placebo.

**Fig. 3:**
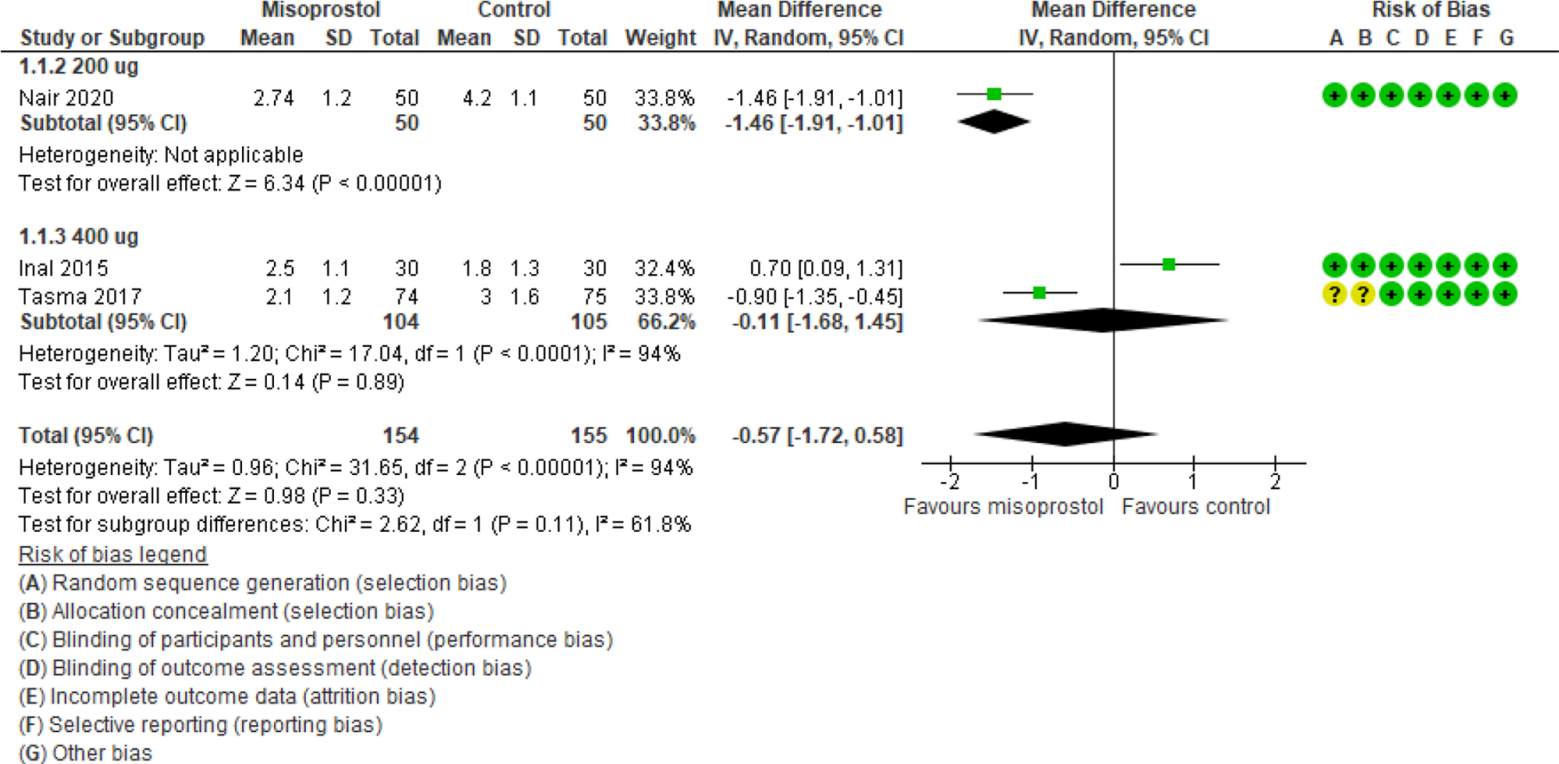
Misoprostol vs placebo ease of dilatation

Ease of cervical dilatation was reported in 3 studies (309 participants) and revealed an effect estimate mean difference of -0.57 with [-1.72, 0.58] 95% CI and a P value of 0.33 and heterogeneity I_2_ 94% (Fig. 3).

**Fig. 4:**
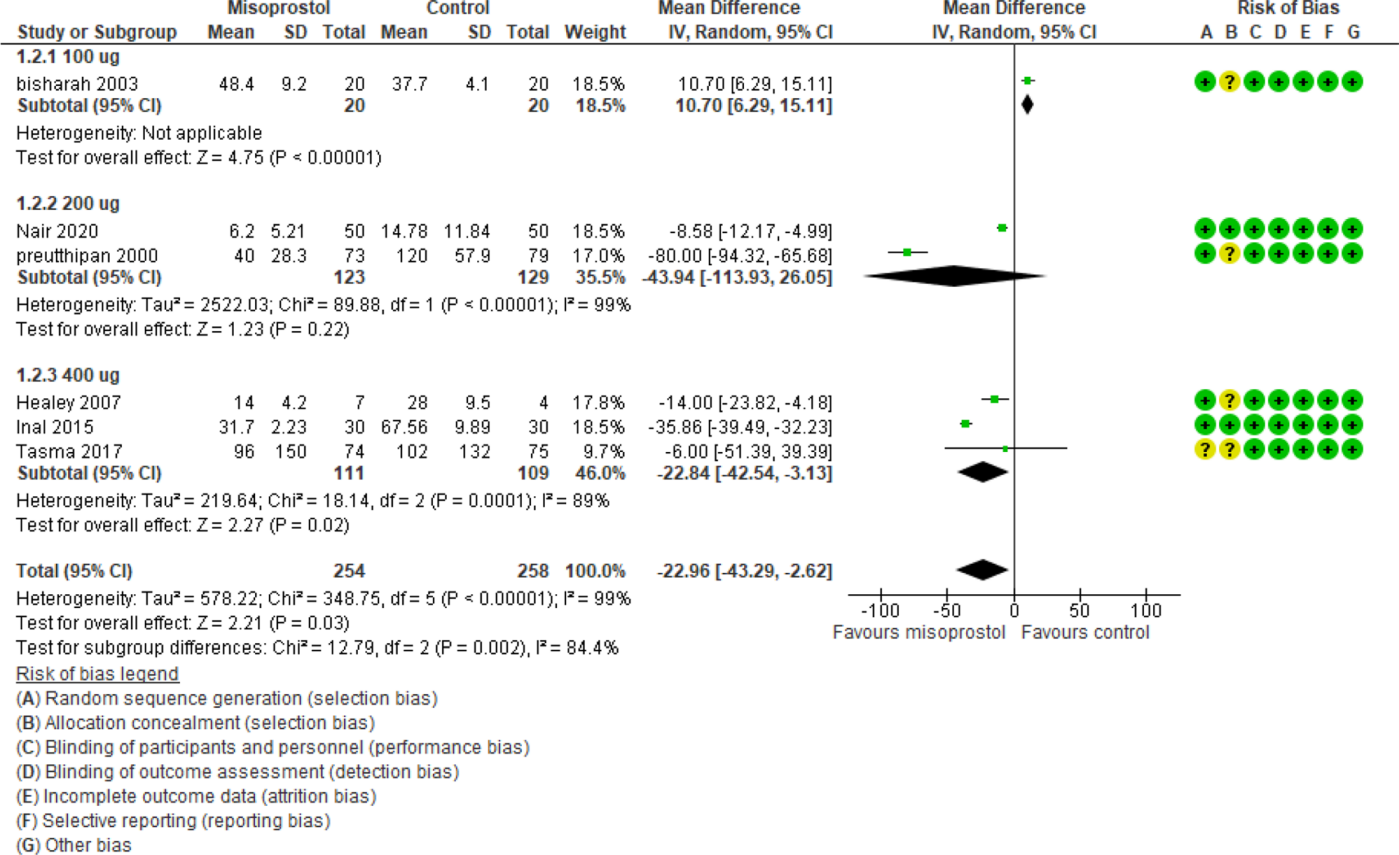
Misoprostol vs placebo time of dilatation

The time needed for cervical dilatation was reported in 6 studies (512 participants) and revealed an effect estimate mean difference of -22.96 min with [-43.29, -2.62] 95% CI and a P value of 0.03 and heterogeneity I_2_ 99% (Fig. 4).

**Fig. 5:**
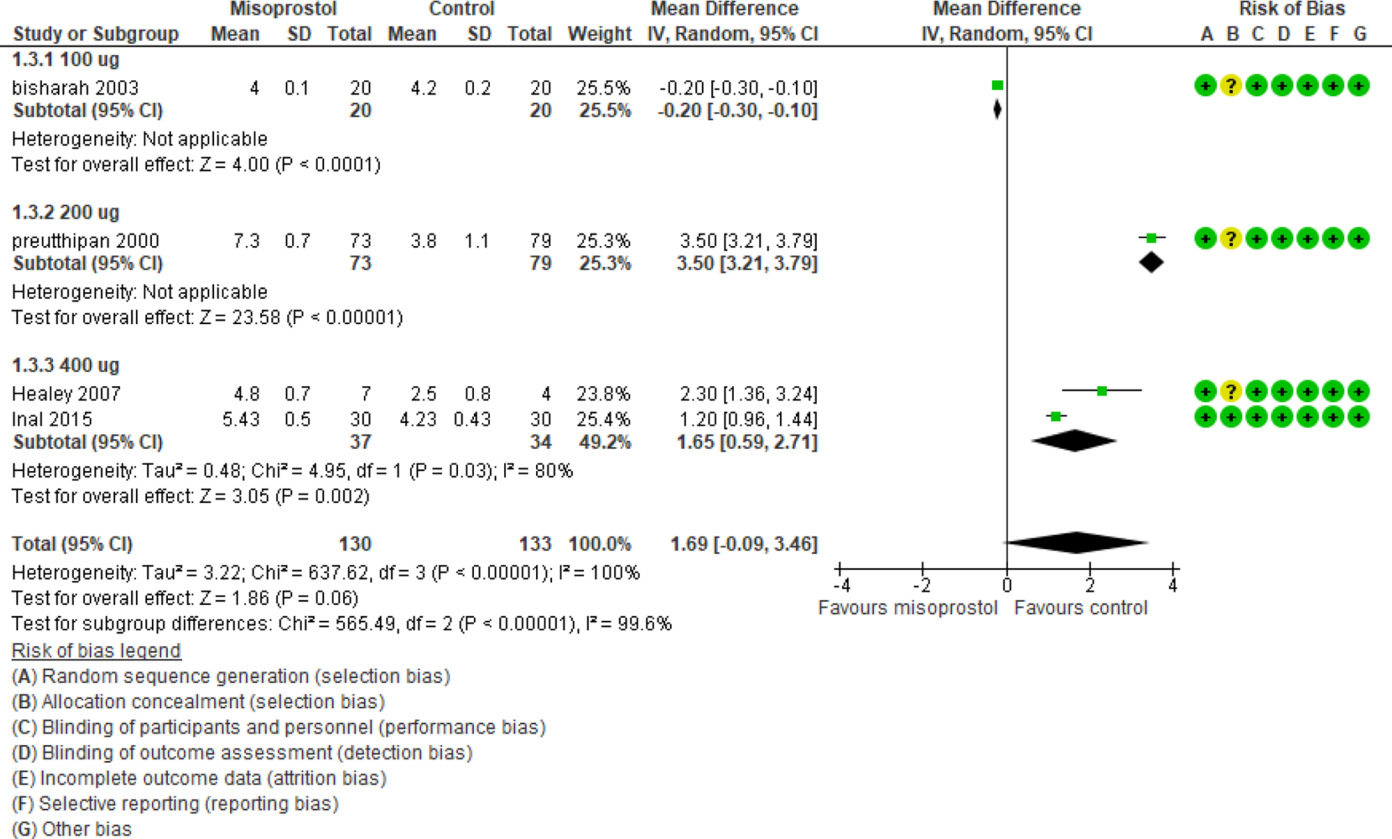
Misoprostol vs placebo preoperative cervical width

The preoperative cervical width was reported in 4 studies (263 participants) and revealed an effect estimate mean difference of 1.69 mm with [-0.09, 3.46] 95% CI and a P value of 0.06 and heterogeneity I_2_ 100% (Fig. 5).

The number of women with failure of cervical dilatation or who needed further dilatation was reported in 4 studies (372 participants) and revealed an effect estimate mean difference of 0.40 with [0.13, 1.17] 95% CI and a P value of 0.09 and heterogeneity I_2_ 66% (figure S1).

The preoperative pain was reported in 3 studies (351 participants) and revealed an effect estimate mean difference of -0.56 with [-2.30, 1.18] 95% CI and a P value of 0.53 and heterogeneity I_2_ 93% (figure S2).

Total number of cases who experienced side effects and procedure complications were reported in 2 and 3 studies (249 and 252 participants) respectively and revealed an effect estimate Odd Ratio of 1.99 and 0.42 with [0.27, 14.67] and [0.14,1.32] 95% CI and a P value of 0.50 and 0.14 and heterogeneity I_2_ 63.7% and 19.5% respectively.

Analysis of specific side effects and specific complications of the procedure was described in Table 3, figures S3 and S4.

Table 3 also shows subgroup analysis of all outcomes according to the dose, route and timing of administration of misoprostol.

**Table 3:** Subgroup analysis of outcomes

Outcome		Subgroup	Studies	Participants	Effect Estimate	*P* value	Heterogeneity
Ease of dilatation	dose	200 ug	1	100	-1.46 [-1.91, -1.01]	< 0.001	NE
		400 ug	2	209	-0.11 [-1.68, 1.45]	0.14	I^2^ 94%, *P* < 0.001
	Route	Oral	1	149	-0.90 [-1.35, -0.45]	< 0.001	NE
		Vaginal	2	160	-0.39 [-2.51, 1.73]	0.72	I^2^ 97%, *P* < 0.001
	Timing	4 h	1	100	-1.46 [-1.91, -1.01]	< 0.001	NE
		6 h	1	60	0.70 [0.09, 1.31]	0.02	NE
		12 and 24 h	1	149	-0.90 [-1.35, -0.45]	< 0.001	NE
Time of cervical dilatation	Dose	100 ug	1	40	10.70 [6.29, 15.11]	< 0.001	NE
		200 ug	2	252	-43.94 [-113.93, 26.05]	0.22	I^2^ 99%, *P* < 0.001
		400 ug	3	220	-22.84 [-42.54, -3.13	0.02	I^2^ 89%, *P* < 0.001
	Route	Oral	2	160	-13.64 [-23.24, -4.05]	0.005	I^2^ 0%, P 0.74
		Vaginal	3	312	-40.33 [-66.69, -13.98]	0.003	I^2^ 99%, *P* < 0.001
		sublingual	1	40	10.70 [6.29, 15.11]	< 0.001	NE
	Timing	4 h	1	100	-8.58 [-12.17, -4.99]	< 0.001	NE
		6 h	1	60	-35.86 [-39.49, -32.23]	< 0.001	NE
		9–10 h	1	152	-80.00 [-94.32, -65.68]	< 0.001	NE
		12 h	2	51	-1.24 [-25.44, 22.95]	0.92	I^2^ 95%, *P* < 0.001
		12 and 24 h	1	149	-6.00 [-51.39, 39.39]	< 0.80	NE
Preoperative cervical width	Dose	100 ug	1	40	-0.20 [-0.30, -0.10]	< 0.001	NE
		200 ug	1	152	3.50 [3.21, 3.79]	< 0.001	NE
		400 ug	2	71	1.65 [0.59, 2.71]	0.002	I^2^ 80%, P 0.03
	Route	Oral	1	11	2.30 [1.36, 3.24]	0.04	I^2^ 99%, *P* < 0.001
		Vaginal	2	212	2.35 [0.09, 4.60]	< 0.001	NE
		sublingual	1	40	-0.20 [-0.30, -0.10]	< 0.001	NE
	Timing	6 h	1	60	1.20 [0.96, 1.44]	< 0.001	NE
		9–10 h	1	152	3.50 [3.21, 3.79]	< 0.001	NE
		12 h	2	51	1.00 [-1.44, 3.45]	0.42	I^2^ 96%, *P* < 0.001
Failure to dilate cervix or need further dilatation	Dose	200 ug	1	152	0.16 [0.05, 0.51]	0.002	NE
		400 ug	3	220	0.60 [0.20, 1.77]	0.35	I^2^ 49%, P 0.14
	Route	Oral	2	160	0.61 [0.08, 4.79]	0.64	I^2^ 55%, P 0.13
		Vaginal	2	212	0.26 [0.11, 0.62]	0.002	I^2^ 18%, P 0.27
	Timing	6 h	1	60	0.40 [0.13, 1.21]	0.10	NE
		9–10 h	1	152	0.16 [0.05, 0.51]	0.002	NE
		12 h	1	11	0.13 [0.01, 2.18]	0.16	NE
		12 and 24 h	1	149	1.26 [0.51, 3.12]	0.62	NE
Preoperative Pain score	Dose	200 ug	2	202	-0.78 [-3.44, 1.89]	0.57	I^2^ 96%, *P* < 0.001
		400 ug	1	149	-0.10 [-1.05, 0.85]	0.84	NE
	Route	Oral	1	149	-0.10 [-1.05, 0.85]	0.84	NE
		Vaginal	2	202	-0.78 [-3.44, 1.89]	0.57	I^2^ 96%, *P* < 0.001
	Timing	4 h	1	100	-2.12 [-2.79, -1.45]	< 0.001	NE
		6 h	1	102	0.60 [-0.27, 1.47]	0.18	NE
		12 and 24 h	1	149	-0.10 [-1.05, 0.85]	0.84	NE
Time of the procedure	Dose	200 ug	3	354	-0.28 [-1.33, 0.78]	0.52	I^2^ 19%, P 0.29
		400 ug	2	209	-0.22 [-0.95, 0.51]	0.56	I^2^ 0%, P 0.68
	Route	Oral	1	149	-0.70 [-3.09, 1.69]	0.57	NE
		Vaginal	4	414	-0.25 [-0.80, 0.31]	0.38	I^2^ 38%, P 0.18
	Timing	4 h	1	100	-0.40 [-0.79, -0.01]	0.04	NE
		6 h	2	162	-0.04 [-0.67, 0.58]	0.89	I^2^ 0%, *P* < 0.58
		9–10 h	1	152	-9.50 [-18.94, -0.06]	0.05	NE
		12 and 24 h	1	149	-0.70 [-3.09, 1.69]	0.07	NE
Side effects	All		2	249	1.99 [0.27, 14.67]	0.50	I^2^ 63.7%, P 0.10
	Nausea		3	311	1.44 [0.67, 3.10]	0.35	I^2^ 0%, P 0.54
	Vomiting		3	311	1.76 [0.56, 5.53]	0.33	I^2^ 12%, P 0.32
	Diarrhea		1	149	2.51 [0.62, 10.10]	0.20	NE
	Pain		3	249	2.12 [0.97, 4.62]	0.06	I^2^ 0%, P 0.41
	Bleeding		3	249	3.25 [1.00, 10.54]	0.05	I^2^ 0%, P 0.67
Complications	All		3	252	0.42 [0.14, 1.32]	0.14	I^2^ 19.5%, P 0.29
	Cervical lacerations		3	252	0.42 [0.09, 1.90]	0.26	I^2^ 38%, P 0.20
	False tract		2	212	0.30 [0.06, 1.52]	0.15	I^2^ 0%, P 0.61
	Uterine perforation		2	212	0.26 [0.03, 2.38]	0.23	I^2^ 0%, *P* < 0.85

Effect estimate was presented as @ mean differences [95% CI] or # Odd Ratio [95% CI]

### Misoprostol versus Dinoglandin

In the 3 studies comparing misoprostol to dinoglandin, The ease of cervical dilatation, time needed for cervical dilatation (figure S5) and preoperative cervical width (figure S6) were evaluated in 1,3 and 2 studies with 60, 436 and 376 participants respectively. The estimated mean differences were not estimated, 0.17 min and 0.01 mm; 95% CI were not estimated, [-4.70, 5.05], and [-0.78, 0.79]; P values of 0.94, 0.98 and 0.99 and I_2_ of 96%,95% and 74% respectively.

The number of women with failure of cervical dilatation, nausea, vomiting, diarrhea, fever, pain, bleeding, cervical lacerations, False tract and uterine perforation had an OR estimate [95% CI] of 0.99 [0.32, 3.06], 1.73 [0.79, 3.76], 1.77 [0.56, 5.57], 2.87 [0.75, 11.03], 4.50 [1.23, 16.42], 2.03 [1.28, 3.22], 2.16 [1.26, 3.70], 0.90 [0.05, 15.25], 0.99 [0.25, 3.92], 0.21 [0.01, 4.31]; and P values of 0.99, 0.17, 0.33, 0.12, 0.02, 0.003, 0.005, 0.94, 0.99 and 0.31 respectively (Figures S7-S9).

### Misoprostol versus misoprostol

In 3 studies different timings of misoprostol administration were compared.

Bakas et al. in 2012 [19] compared 39 women who received 200 µg of oral misoprostol twice at 12 and 6 h and 36 women who received 200 µg of vaginal misoprostol 12 h to 35 women who received 200 µg of vaginal misoprostol 4 h before hysteroscopy. They reported a lower number of women who needed cervical dilatation and a shorter time for cervical dilatation in the 1st 2 groups compared to the 3rd group (12.8% vs. 16.6% vs.74.1%; *P* < 0.001, and 35.3 ± 18 vs. 37.5 ± 21 vs. 63.7 ± 23 s; *P* < 0.001, respectively) with no significant differences regarding the drug side effects between the 3 groups. They recommended the use of the 1st 2 protocols over the 3rd one.

Fouda et al. in 2016 [22] compared 60 women who received 400 µg of vaginal misoprostol 12 h to 60 women who received the same dose 3 h before office hysteroscopy. They reported a significantly lower pain score during hysteroscopy (37.98 ± 13.13 vs. 51.98 ± 20.68; *P* < 0.001), easier passing through the cervical canal (48.9 ± 17.79 vs. 58.28 ± 21.85; *P* = 0.011) and significantly not different pain score recorded 30 min after hysteroscopy (11.92 ± 7.22 vs. 13.3 ± 6.73; *P* = 0.28) in the long-interval group compared to short interval group.

Mohamed et al. 2020 [26] compared 3 groups of women (each has 66 participants) who received 400 ug of vaginal misoprostol at 12, 6 and 3 h before hysteroscopy. They found significant differences between the 12,6 and 3 h groups regarding Pain VAS score (2.6 ± 1.3; 5.3 ± 1.3 and 7.3 ± 1.2, *P* < 0.001), Ease of cervical dilatation (4.2 ± 0.7 3,0.5 ± 0.5 and 2.5 ± 0.6, *P* < 0.001), preoperative cervical width (5.9 ± 0.8, 4.7 ± 1.1 and 3.9 ± 0.8, *P* < 0.001) and Case acceptability (4.2 ± 0.7, 3.5 ± 0.5 and 2.5 ± 0.6, *P* < 0.001) respectively.

Batuken eta al [20] compared 39 (19 nullipara) women who received 400 ug of oral misoprostol to 38 (21 nullipara) women who received 400 ug of vaginal misoprostol. In nullipara, there was a significant difference regarding preoperative cervical width (5.6 ± 1.5 vs. 6.7 ± 1.5; *P* = 0.016), and significant difference regarding time needed for cervical dilatation (111.5 ± 113.5 vs. 55.8 ± 38.0 *P* = 0.049), number of women who need cervical dilatation (21 (100%) vs. 15 (78.9%), *P* = 0.042) in the oral and vaginal group respectively.

No meta-analysis was done for these studies as they have marked discrepancies in routes, dose and timing of administration.

### Misoprostol versus diclofenac

Hassa et al. [23] compared 51 women who received 200 ug of vaginal misoprostol 6 h before outpatient hysteroscopy to 50 women who received 100 mg of rectal diclofenac sodium 1 h before the procedure. They found no significant differences between the 2 groups regarding pain scores [6.7 (4.6–8.8) vs. 6.2 (3.0–7.6)], patient acceptance [3.13 (2.52–4.42) vs. 2.91 (2.30–3.87)], vasovagal symptoms [3 (5.4) vs. 2 (4)], procedure time [3.1 (2.5–3.7) vs.2.8 (2.3–3.5)], and postprocedural analgesic requirement [2 (3.6) vs.1 (2)] respectively with P values > 0.05.

## Discussion

### Main findings

Thirteen RCTs that compared misoprostol administration to placebo, dinoglandin or nonsteroidal anti-inflammatory drug before hysteroscopy in nulliparous women were included in this systematic review. The dose or misoprostol ranged between 100 and 400 ug administered through oral, vaginal or sublingual routes and the timing of its intake before hysteroscopy ranged between 3 and 24 h.

The pooled evidence showed that preoperative administration of misoprostol in nulliparous women is associated with a significant reduction in the time needed for cervical dilatation. This effect was evident in the 100 and 400 ug dose group, through all studied routes and at 4,6,9–10 h before the procedure and a fair non-significant reduction in the number of failures (*P* = 0.09) and cases needing further dilatation and non-significant wider preoperative cervical width (*P* = 0.06) when compared to placebo administration. While the effect estimate could not find any significant difference between the 2 groups regarding Ease of cervical dilatation (*P* = 0.33), preoperative pain score (*P* = 0.53), Total number of cases who experienced side effects(*P* = 0.50), specific side effects (nausea, vomiting, diarrhea, pain and bleeding), total number of complications (*P* = 0.14), specific complications (cervical laceration, false tract and uterine perforation). The absence of significant differences among these outcomes may be related to the small sample size of most of the included studies so that each individual study failed to reach a significant value. Recalculation of sample size considering these outcomes in future studies may confirm these differences.

Apart from the significantly higher number of cases who experienced fever, preoperative pain and bleeding after taking misoprostol, there was no significant difference regarding the time needed for cervical dilatation, preoperative cervical width, the number of women with failure of cervical dilatation, nausea, vomiting, diarrhea, procedure complications as cervical lacerations, creation of false tract and uterine perforation between women with misoprostol and those with dinoglandin administration.

Misoprostol has many advantages over dinoglandin being inexpensive, easily storable, available drug that can be used through any mucous membrane (oral, vaginal, rectal, sublingual, and intrauterine [10].

The effect of prostaglandins and their analogues on cervical ripening and dilatation is achieved through degradation of connective tissue collagen of the cervical stromal and enhancement of uterine contractility. Although misosprostol bind [14].

These effects occur through binding to E prostanoid receptors named from 1 to 4 where EP 1 and 3 increase and EP2 and 4 decrease smooth muscle contractility. Misoprostol can bind both EP 2 and 3 receptors with higher affinity to the EP3 type. This binding causes uterine contractions and cervical relaxation (ripening) [31].

According to recent evidence, the hormonal pre-operative before hysteroscopic surgery may offer a clearer view of the uterine cavity and, in this way, reduce the operative time and even complication rate.

### Strengths and limitations

This is the first systematic review that evaluate the value of misoprostol administration before hysteroscopy in nulliparous women. Some previous reviews studied misoprostol value before hyteroscopy in women without any specification of characteristics of these women regarding reproductive status, parity or menopause. As the main difficulties and complications during outpatients’ procedures as hysteroscopy are mainly related to cervical dilatation, women with certain cervical status as nullipara, menopausal women and those with cervical stenosis need special considerations as Nullipara and postmenopausal women are more susceptible to experience pain and other complications of hysteroscopy as these women have less elastic and less dilated cervical os [9].

We conducted a systematic review in menopausal women [14] and this one was conducted on nulliparous women. As the number of nullipara is progressively increasing and the use of hysteroscopy especially as an outpatient procedure is also increasing, the conduction of this review was essential. Thirteen RCTs represent all the published and unpublished studies reached by comprehensive searching of all available sources. Proper subgroup analysis according to the different comparators, various doses, routes and timing of misoprostol administration before the procedure was done.

The main limitation of this review is the relatively small number of RCTs conducted on nulliparous women and the marked heterogeneity detected among these studies. The included studies used diversity of doses administered though different routes at different timings of before the procedure. The inconsistent protocols of the studies through using different types of hysteroscopies with different diameters and different indications (diagnostic or operative) with different distension media with or without anaesthesia. We used the random effect model to compensate for this marked heterogeneity beside subgroup analysis for all possible variables. However, subgroup analysis for certain variables as different hysteroscopy types, diameters, indications and the used distension media could not done based on very limited numbers of studies considering these variables. Also, subgroup analysis according to menopausal status of the included participants cannot be done as it was not reported by most of the included studies.

The availability of registration in 3 studies only may raise some issues about potential bias in other studies findings.

### Comparison with Existing Literature

Some systematic reviews conducted to assess the value of misoprostol before hysteroscopy [8, 16, 32, 33]. No single systematic review was focused on its administration in nulliparous women. Al-Fozan and colleagues conducted a Cochrane review to compare the effects of misoprostol versus placebo, dinoglandin and osmotic dilators. Their review included 19 studies and conducted subgroup analysis based on menopausal status but did not consider parity status. Zhuo et al., in 2016 included 32 studies that compared misoprostol to placebo only. There were no specific participants inclusion criteria, and a small subgroup analysis was based only on menopausal condition but also did not consider parity status.

## Conclusions

This systematic review confirmed beneficial effects of misoprostol over placebo on the time needed for cervical dilatation (moderate evidence), but failed to prove any beneficial effects on the preoperative cervical width (moderate evidence), number of failure of cervical dilatation or cases needing further dilatation (moderate evidence), Ease of cervical dilatation (low evidence), preoperative pain score (moderate evidence), Total number of cases who experienced side effects (low evidence), specific side effects (nausea (moderate evidence), vomiting (moderate evidence), diarrhea (very low evidence), pain (high evidence) and bleeding (high evidence), total number of complications (moderate evidence), specific complications (cervical laceration (low evidence), false tract (moderate evidence) and uterine perforation (moderate evidence)).

High evidence findings suggest and confirm the drug use while moderate and low evidences means that a more supporting trials and data are needed to support its use.

The reduction of time needed for cervical dilatation is beneficial to reduce the operative time, exposure to anesthetic agents (if any) with the resultant reduction of the procedure costs. It allow more procedures to be conducted as an outpatient ones limiting the need for hospital admission which is an important issue especially in limited resource counties.

When misoprostol was compared to dinoglandin, there was no significant difference regarding the time needed for cervical dilatation, preoperative cervical width, the number of women with failure of cervical dilatation, nausea, vomiting, diarrhea, procedure complications as cervical lacerations, creation of false tract and uterine perforation. However, women administered misoprostol reported more preoperative pain and bleeding.

We recommend a future conduction of a well organized double blind RCT with properly calculated sample size and selection of the proper dose and timing of misoprostol administration before hysteroscopy in these particularly high risk women.

## Electronic supplementary material

Below is the link to the electronic supplementary material.

Supplementary Material 1

## Data Availability

Data used and/or analised during the study are available from the corresponding author upon reasonable request.
