# Supplementary material for: The use of misoprostol before hysteroscopy in Nulliparous women: a systematic review and meta-analysis of randomized controlled trials
Source: BMC Pregnancy Childbirth. 2024 Nov 27;24:796. doi: 10.1186/s12884-024-06993-z (PMC11600905; doi:10.1186/s12884-024-06993-z)
Supplement: Supplementary file 1 — Supplementary Material 1 [file 12884_2024_6993_MOESM1_ESM.docx]

Supplementary figures


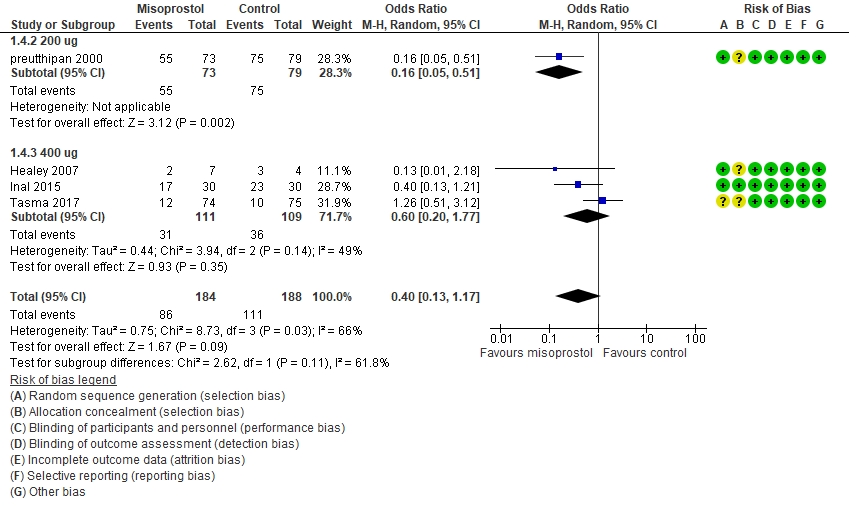


Figure S1 Misoprostol vs placebo Failure to dilate cervix or need further dilatation


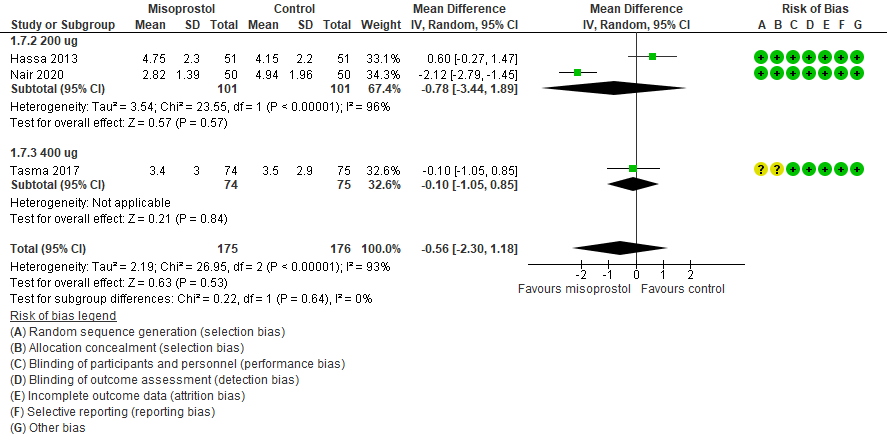


Figure S2 Misoprostol vs placebo preoperative pain score


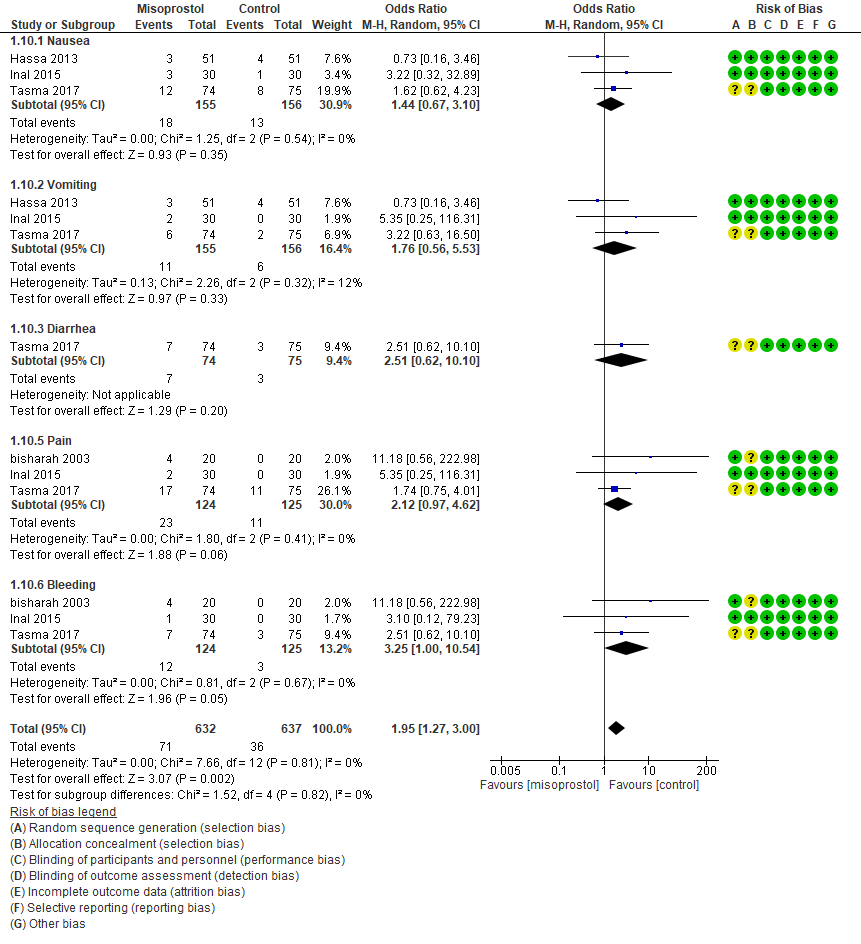


Figure S3 Misoprostol vs placebo Specific side effects

**
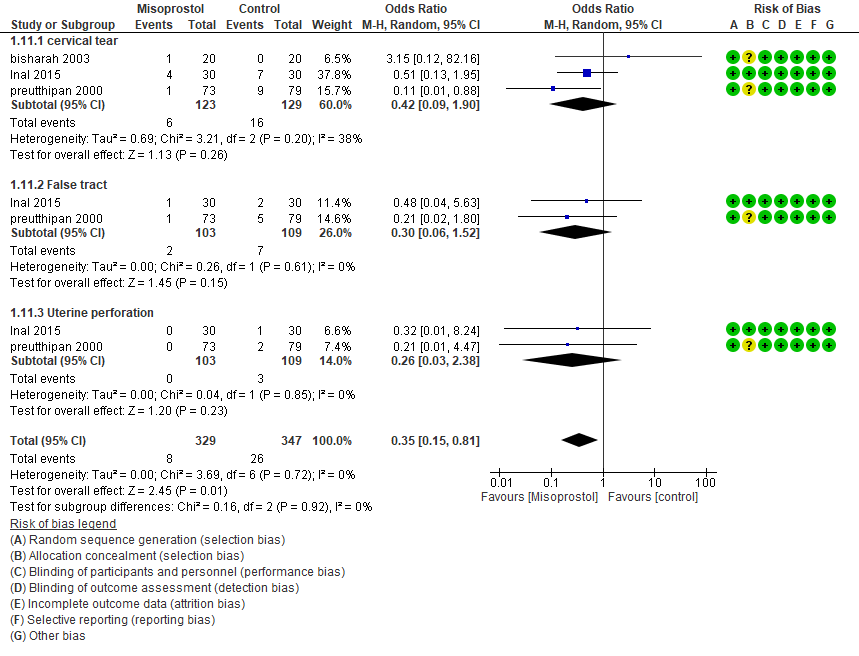
**

Figure S4 Misoprostol vs placebo Specific complications


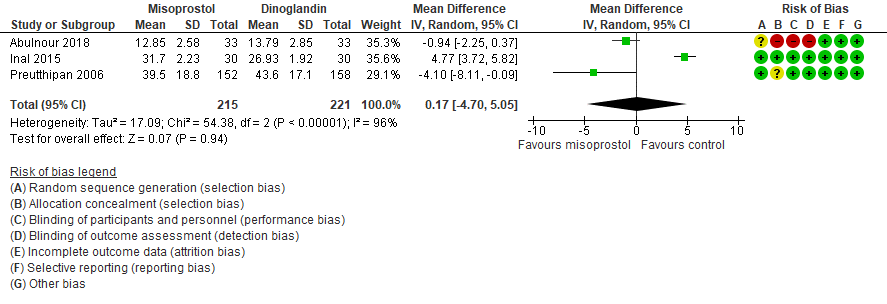


Figure S5 Misoprostol vs Dinoglandin Time of cervical dilatation


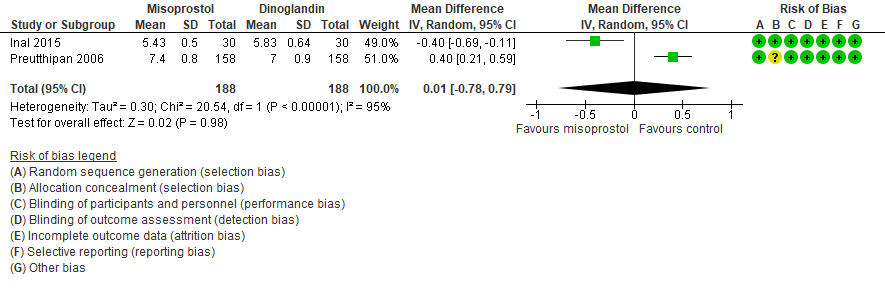


Figure S6 Misoprostol vs Dinoglandin preoperative cervical width


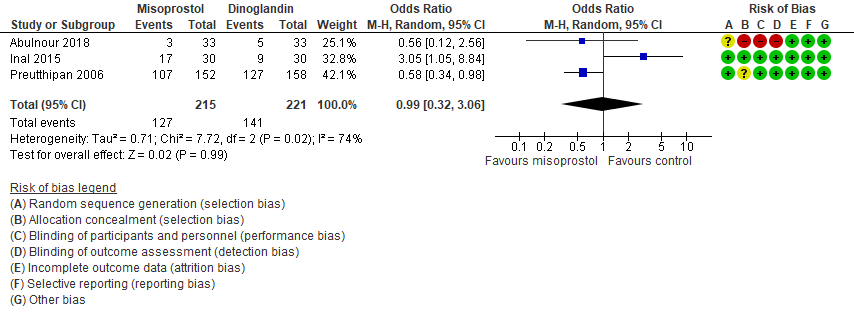


Figure S7 Misoprostol vs Dinoglandin Failure to dilate cervix or need further dilatation


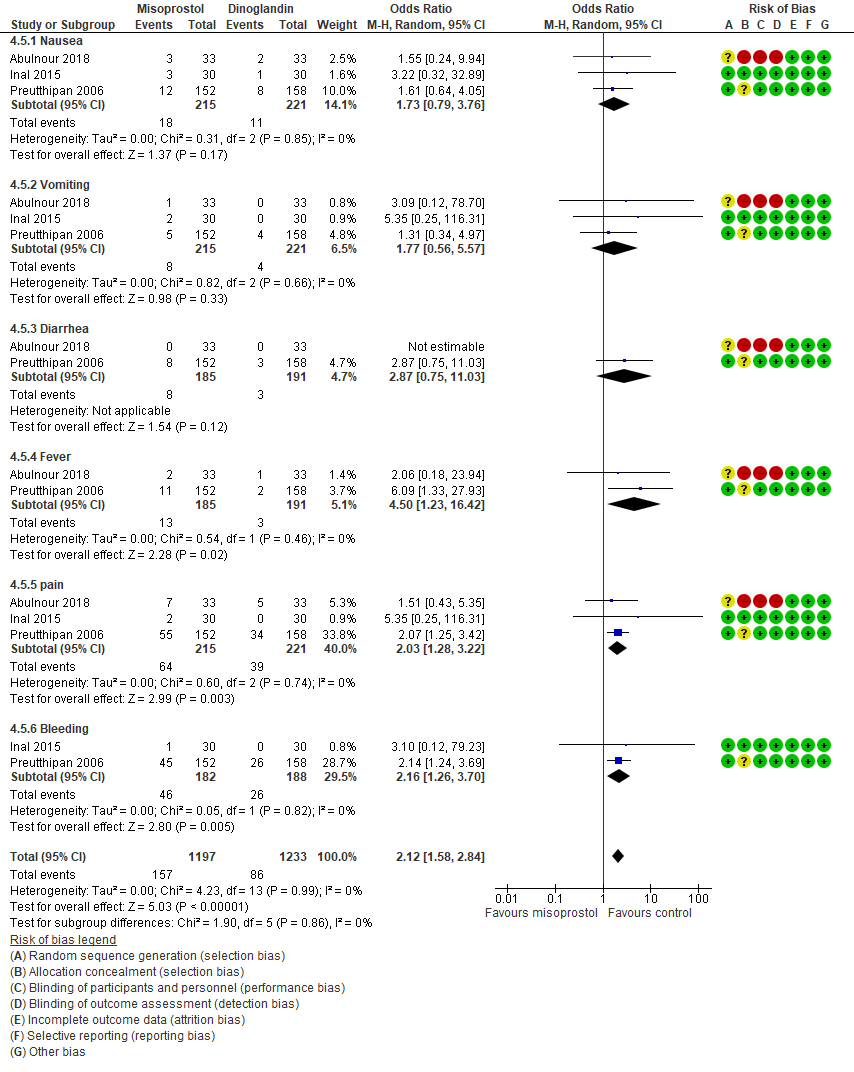


Figure S8 Misoprostol vs Dinoglandin Specific side effects


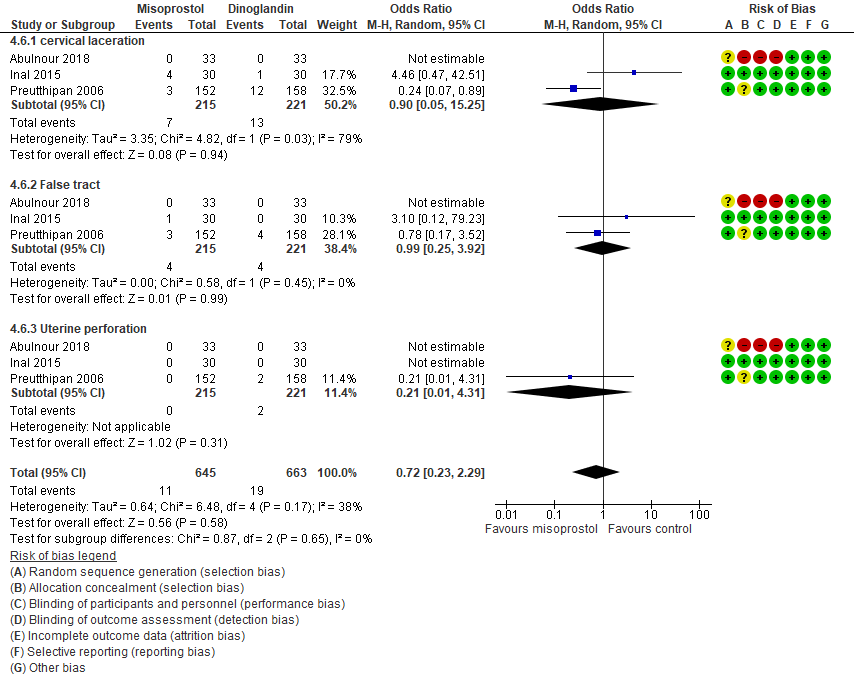


Figure S9 Misoprostol vs Dinoglandin Specific complications
